# Tired Mothers

**Published:** 1884-04

**Authors:** 


### TIRED MOTHERS.

MANY a Mother grows old, faded and feeble long before her time, because her boys and girls are not thoughtfully considerate and helpful When they become old enough to be of service in a household, mother has become so used to doing all herself; to taking upon her shoulders all the care, that she forgets to lay off the burden little by little, upon those who are so well able to bear it.

